# Evaluation of Hyperspectral Imaging for Follow-Up Assessment after Revascularization in Peripheral Artery Disease

**DOI:** 10.3390/jcm11030758

**Published:** 2022-01-30

**Authors:** Eberhard Grambow, Niels Arne Sandkühler, Justus Groß, Daniel G. E. Thiem, Michael Dau, Matthias Leuchter, Malte Weinrich

**Affiliations:** 1 Department of General, Visceral, Thoracic, Vascular and Transplantation Surgery, Rostock University Medical Center, 18057 Rostock, Germany; niels.s29@googlemail.com (N.A.S.); justus.gross@med.uni-rostock.de (J.G.); matthias.leuchter@med.uni-rostock.de (M.L.); 2 Department of Oral and Maxillofacial Surgery, Facial Plastic Surgery, University Medical Centre Mainz, Augustusplatz 2, 55131 Mainz, Germany; daniel.thiem@unimedizin-mainz.de; 3 Department of Oral, Maxillofacial Plastic Surgery, Rostock University Medical Center, 18057 Rostock, Germany; michael.dau@med.uni-rostock.de; 4 Department for Vascular Medicine, DRK Kliniken Berlin Köpenick, 12559 Berlin, Germany; m.weinrich@drk-klinik-berlin.de

**Keywords:** TIVITA^®^ Tissue, tissue oxygenation, microcirculation, peripheral artery disease, perfusion monitoring

## Abstract

Background: Assessment of tissue oxygenation is an important aspect of detection and monitoring of patients with peripheral artery disease (PAD). Hyperspectral imaging (HSI) is a non-contact technology for assessing microcirculatory function by quantifying tissue oxygen saturation (StO_2_). This study investigated whether HSI can be used to monitor skin oxygenation in patients with PAD after appropriate treatment of the lower extremities. Methods: For this purpose, 37 patients with PAD were studied by means of ankle–brachial index (ABI) and HSI before and after surgical or endovascular therapy. Thereby, the oxygenation parameter StO_2_ and near infrared (NIR) perfusion index were quantified in seven angiosomes on the diseased lower leg and foot. In addition, the effects of skin temperature and physical activity on StO_2_ and the NIR perfusion index and the respective inter-operator variability of these parameters were investigated in 25 healthy volunteers. Results: In all patients, the ABI significantly increased after surgical and endovascular therapy. In parallel, HSI revealed significant changes in both StO_2_ and NIR perfusion index in almost all studied angiosomes depending on the performed treatment. The increase in tissue oxygenation saturation was especially pronounced after surgical treatment. Neither heat nor cold, nor physical activity, nor repeated assessments of HSI parameters by independent investigators significantly affected the results on StO_2_ and the NIR perfusion index. Conclusions: Tissue oxygen saturation data obtained with HSI are robust to external confounders, such as temperature and physical activity, and do not show inter-operator variability; therefore, can be used as an additional technique to established methods, such as the ABI, to monitor peripheral perfusion in patients with PAD.

## 1. Introduction

Peripheral artery disease (PAD) is a common vascular disease that affects more than 200 million people worldwide [1,2,3]. It is most often caused by arterial atherosclerosis [4,5]. Subsequent hemodynamic alterations leading to hypoxia can trigger a cascade of events leading to macro- and microvascular changes in the affected limb [6]. National and international guidelines recommend surgical or endovascular treatment with respect to physical alterations, i.e., reduced walking distance due to pain, pain even in a resting state or non-healing wounds [5,7]. Common diagnostic modalities for PAD include the ankle–brachial index (ABI) [8], toe pressure measurement and duplex ultrasound (DUS) analysis, as well as computed tomography (CT) or magnetic resonance (MR) angiography and digital subtraction angiography [7]. None of these techniques provides information on regional perfusion of the limb. While DUS is limited by the experience of the examiner and by anatomical aspects of the studied individual (arterial wall calcification, tissue edema, thickness of subcutaneous adipose tissue), CT and MR angiography need radiation (CT), contrast agents (CT and MR), have long examination times (MR) and are not feasible for patients with magnetic implants (MR) or claustrophobia (MR more than CT).

Novel techniques for the quantification of reginal volumetric limb perfusion further include quiescent-interval single-shot (QUISS) MR angiography, an unenhanced, electrocardiograph-gated, two-dimensional, balanced, steady-state, free-precession technique [9,10], and radiotracer-based single photon emission computed tomography (SPECT) or positron emission tomography (PET) in combination with both CT and MR [11]. Thereby, SPECT/CT and PET/CT can be used to study the pathophysiology within lower extremity vasculature and skeletal muscle molecular processes in patients with PAD. Since both radiotracer-based imaging techniques do not need nephrotoxic contrast agents, they are also feasible for patients with impaired kidney function [11]. Although these novel molecular imaging strategies are capable of detecting underlying pathologies in PAD, they still bear the disadvantages of radiation being admitted to the patients, which limits routine use for follow-up, and of being cost-intensive and, therefore, are not available for the variety of patients with PAD. In parallel to QUISS, QUISS-arterial, spin-labelled MR angiography is a QUISS-related subtraction-based technique [12]. Both techniques are feasible for detecting arterial stenosis in PAD without the need for contrast agents [9] but are actually not used for routine perfusion imaging in patients with PAD.

In recent years, hyperspectral imaging (HSI) has emerged in the field of vascular medicine. It is a non-invasive, non-contact imaging technique that enables spectroscopical measurement of tissue oxygenation without the need for contrast agents or radiation [13]. Although the feasibility of HSI in the detection of impaired perfusion in patients with peripheral artery disease (PAD) has already been shown [14,15,16], none of the existing HSI systems gained significance for routine, clinical use. Our group recently proved that hyperspectral imaging can be an important, additional tool to support and improve the diagnosis of PAD. In this study, we underlined the feasibility of HSI for fast and reliable detection of impaired tissue perfusion in patients with PAD [17].

The aim of this study was to evaluate whether HSI analysis is feasible for detecting changes in tissue oxygen saturation after surgical and endovascular revascularization and, therefore, whether it could be used for the monitoring of patients with PAD. In addition, the effects of temperature, physical activity and of different investigators on the HSI analysis were addressed in healthy individuals.

## 2. Materials and Methods

The study was conducted from January 2019 to December 2020. Written, informed consent of all patients and healthy volunteers was obtained before recruitment into the study, in accordance with the vote of the local ethics committee (A 2017-0153). Exclusion criteria were: age younger than 18 years, a history of minor or major amputation at the lower extremities, inability to consent, tattoos or pigment alteration on the limbs or colonization with multi-resistant pathogens.

Initially, 25 healthy volunteers aged 18 to 35 years, who had no history of vascular disease or previous lower limb surgery and were not taking any medication, were studied to test the robustness of HSI data in terms of temperature, physical activity and inter-operator variability. Then, 37 patients with PAD, who were hospitalized for surgical or endovascular therapy, were included in this prospective single-center study. The presence of PAD was established using the criteria of an ABI < 0.9 [8], by means of duplex ultrasound, as well as by CT or MR angiography.

Data assessment followed a standardized protocol and was performed in the same room to assure standardized light conditions and an ambient room temperature (20–23 °C). Except for analysis of inter-operator variability, one of the authors (N.A.S.) performed all HSI studies and data analysis and was not blinded. For analysis of temperature, physical activity and inter-operator variability, volunteers were required to abstain from smoking for at least one hour prior to data assessment. After written, informed consent was obtained, all study participants were first asked for their medical history, medication and prior vascular interventions or operations. Stable conditions in blood pressure and heart rate were reached easily in all participants during this 10 min procedure. To assess the robustness of the HSI data with respect to temperature, healthy subjects were required to take either a cold (with crushed ice, approximately 4 °C) or a warm foot bath of approximately 45 °C for five minutes each. The effect of physical activity on HSI data was assessed after 50 repetitions of a rocking-to-the-toe stand. To avoid mutual influence of a hot or cold foot bath and physical activity, the respective experiments were performed on different days. For assessment of inter-observer variability, HSI analysis was performed by three independent investigators. Since only the feet were affected by the temperature and referring to our previous study [17], HSI assessment was performed only in the angiosome of the medial plantar artery of both feet, with the volunteer laying in prone position on a standard hospital stretcher.

**Figure 1:**
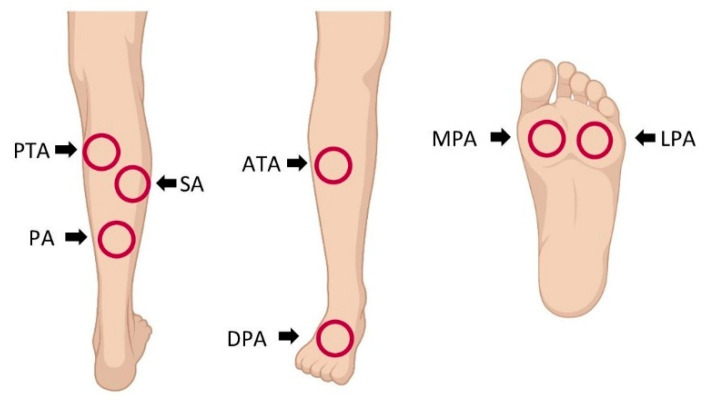
Locations for hyperspectral imaging (HSI) assessment in patients with peripheral artery disease: HSI was performed in the angiosomes of the posterior tibial artery (PTA), sural artery (SA), peroneal artery (PA), anterior tibial artery (ATA), dorsal pedal artery (DPA), medial plantar artery (MPA) and lateral plantar artery (LPA).

Compared to HSI measurement in healthy volunteers, analysis in patients with PAD was performed in seven angiosomes [18] in the diseased lower limb (Figure 1).

After HSI analysis on the day of hospitalization, the skin temperature within the angiosome was measured with an infrared thermometer (Metene Digital Infrared Non-Contact Forehead Thermometer, Metene, Meilong Town, Longhua District, Shenzhen, China), followed by ABI quantification (VascAssist^®^, iSYMED, Butzbach, Germany) [19]. Afterwards, complaint-free walking distance was measured in a standardized manner. In surgically treated patients, HSI and ABI were additionally measured on the first, third and fifth day after surgery. Patients treated endovascularly were discharged on the first day after intervention; therefore, HSI and ABI were measured only once, on the first day after intervention.

The CE-certified TIVITA^®^ Tissue camera (Diaspective Vision, Pepelow, Germany) was used for all HSI analyses. The camera system and the underlying technique for hyperspectral analysis have already been described in detail [17]. HSI analysis includes the parameters tissue oxygenation saturation (StO_2_), near infrared (NIR) perfusion index, tissue hemoglobin index (THI) and tissue water index (TWI). The parameters StO_2_ and NIR perfusion index describe the relative oxygen saturation of the blood in the microcirculatory system of the examined tissue area from different tissue depths. The THI describes the existing hemoglobin distribution in the microcirculatory system of the examined tissue area. The TWI has the same meaning in relation to water.

The calculation algorithms for these are based on the specific absorption peaks of hemoglobin HHb, oxygenated hemoglobin O_2_Hb and water. StO_2_ is calculated by using the second derivative from the spectrum. With this step, constant and linear influences are eliminated, and absorption bands are amplified. Since the absorption curves of melanin and scattering are nearly linear in the interesting wavelength range, this step reduces the influence of scattering and melanin on the calculated values. NIR perfusion index, THI and TWI are based on the recorded absorbance spectrum, whereby individual wavelength ranges are evaluated for each parameter.

From a theoretical point of view and the suggestion of light path simulations, the remission spectroscopic measurement in the visible range (500–650 nm) gathers information about the superficial tissue layers, whereas the measurement in the NIR range (650–1000 nm) provides information about deeper tissue layers. Based on the well-known optical properties of human skin, a typical light penetration depth can be estimated to be between approximately 0.8 mm (500 nm) and 2.6 mm (1000 nm) [20,21]. Because the StO_2_ calculation is based on the VIS spectral range, it displays the oxygenation of the skin at approximately 0.8 mm depth, and the parameter NIR perfusion index allows evaluation of subcutaneous perfusion at about 2.6 mm depth. It must be noted that a depth-selective measurement is not possible and that all layers penetrated by the light contribute to the signal.

Numerical assessment of these parameters was performed using the camera-specific software package TIVITA^®^ Suite that allows definition of different regions for parameter analysis within the photographed tissue. The parameter calculation and validation were recently explained in more detail [22].

The parameters that are used for statistical representation were calculated in specified circular areas by the software package. The position of the circled area and its diameter could be chosen freely. Upon positioning the marker, the average of each marker parameter was displayed with the respective spectrum. To standardize the quantification of the parameters, the circulated area was always positioned in the center of the angiosome, and the diameter was unchanged between the studied patients.

### Statistics

Statistical analysis was performed using R software (R Core Team, Auckland, New Zealand, version 3.5.2) [23]. Variables were evaluated for normal distribution employing the Shapiro–Wilk test. In case of normal distribution, the *t*-test was used, otherwise a Wilcoxon rank-sum test was used to compare the two groups. Percentages were evaluated using either the chi-square test or the Fisher exact test. For detection of differences between all three groups, a Kruskal–Wallis rank-sum test was performed. Intra-class correlation coefficients ICC(3,k) for normally distributed data were calculated, otherwise Kendall’s concordance coefficient (W) was used to assess the reliability. We assumed that ICC values greater than 0.9 indicated excellent reliability [24]. The non-parametric Skillings–Mack test, as an extension of the Friedman test, was used for repeated measures analysis to compare more than two groups, otherwise the Wilcoxon signed-rank test was used. Hyperspectral parameters were correlated with ABIs and complaint-free walking distances using a Pearson (for normally distributed variables) or Spearman (in case of failed normal distribution) bivariate linear correlation analysis. Data were summarized as mean and 95% confidence interval (skin temperature, StO_2_, NIR perfusion index, TWI and THI) or as median and interquartile range (IQR) (age, pack years, ABI and complaint-free walking distance). Statistical significance was set at *p* < 0.05.

## 3. Results

Six seconds were required for the acquisition of each hyperspectral data cube. During this time, the patient must lie still, and the camera should not be moved to avoid motion artifacts. The software then required a further ten seconds to display the parameters.

### 3.1. Characteristics of Participants

The general characteristics of the healthy volunteers and patients with PAD are given in Table 1. Only symptomatic patients with PAD are included, of whom 65% suffered from claudication (Fontaine stadium IIa and IIb) and 49% from critical limb ischemia (Fontaine stadium III and IV).

### 3.2. Effects of Temperature and Physical Activity on HSI Data

**Figure 2:**
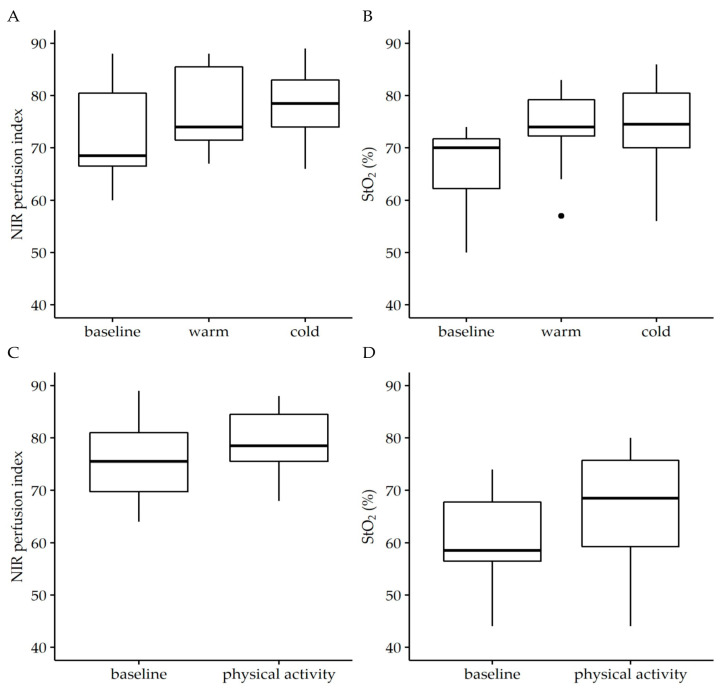
Effect of temperature (**A**,**B**) and physical activity (**C**,**D**) on near infrared perfusion (NIR) perfusion index and tissue oxygenation saturation (StO_2_). The analysis was performed in the angiosome of the medial plantar artery. To simulate differences in external temperature, individuals were asked to take a hot or cold footbath for 5 min before hyperspectral imaging was performed. Physical activity was induced by 50 repetitions of a rocking-to-the-toe stand. Temperature affected neither StO_2_ (W = 0.535, *p* = 0.11) nor NIR perfusion index (W = 0.731, *p* = 0.02) and nor did physical activity (StO_2_: W = 0.646, *p* = 0.235; NIR perfusion index: W = 0.713, *p* = 0.170) relevantly affect the parameters of NIR perfusion index and StO_2_. Wilcoxon signed-rank test. Data are given as box plots indicating the median with the 25th and 75th percentiles. *n* = 25.

Neither extreme temperatures, induced by cold or hot foot bath, nor physical activity, induced by 50 repetitions of a rocking-to-the-toe stand, had a significant effect on the NIR perfusion index or StO_2_ (Figure 2). Median baseline values of the NIR perfusion index and StO_2_ of 68.5 (66.5, 80.5) and 70% (62.3%, 71.75%) were only slightly affected by both the warm foot bath (74 (71.5, 85.5), *p* = 0.606 and 74% (72.3%, 79.3%), *p* = 0.146) and cold foot bath (78.5 (74, 83), *p* = 0.177 and 74.5% (70%, 80.5%), *p* = 0.124), respectively. In parallel, median baseline values of the NIR perfusion index and StO_2_ of 75.5 (69.8, 81) and 58.5 (56.5, 67.8) were not affected by physical activity (78.5 (75.5, 84.5), *p* = 0.358 and 68.5% (59.3%, 75.8%), *p* = 0.202), respectively.

### 3.3. Inter-Operator Variability

Assessment of the NIR perfusion index and StO_2_ in the angiosome of the medial plantar artery in the same individual by three independent investigators revealed no statistically significant differences. The StO_2_ and NIR perfusion index ranged from 66 to 73 (*p* = 0.368, ICC(3) = 0.99 (95% CI: 0.98,1)) and 84 to 93 (*p* = 0.156, ICC(3) = 0.85 (95% CI: 0.74,1)), respectively.

### 3.4. Data of Surgically Treated Patients

The performed surgical treatment in 16 legs of 15 patients included one aorto-bi-femoral bypass, three femoropopliteal bypasses above and six below the knee and three local thrombendarteriectomies of the femoral bifurcation, as well as two thrombendarteriectomies in combination with implantation of a saphenous vein graft below the knee. All patients were discharged from hospital on the fourth to sixth postoperative day. While all 16 patients were be studied on days 0 and 1, only 10 of 16 patients could be studied on day 3 after the operation.

The median ABI in surgically treated patients significantly increased postoperatively to 0.83 (0.78–0.96) and 0.86 (0.72–0.96) on day one and three after the operation when compared to a preoperative value of 0.45 (0.4–0.87, *p* < 0.05).

In parallel, the NIR perfusion index significantly increased in five of the seven studied angiosomes of the foot and the lower limb on day three compared to the preoperative assessment (Table 2). In the angiosome of the dorsal pedal artery, a significant increase of the NIR perfusion index was also found on the first postoperative day. In contrast, StO_2_ was found decreased in all four angiosomes of the calf and markedly increased in the angiosomes of the foot on the first and third postoperative day (Table 2).

Although the ABI and both the NIR perfusion index and StO_2_ increased after the operation, significant correlations between the changes in ABI and StO_2_ were only found in the angiosome of the dorsal pedal artery (R = 0.62, *p* = 0.0011) and posterior tibial artery (R= 0.68, *p* = 0.0036) after surgical revascularization. No studied angiosome revealed a significant correlation between the ABI and NIR perfusion index.

TWI, a marker for tissue edema, significantly increased on day 3 in six of the seven studied angiosomes when compared to the preoperative value (Table 2).

### 3.5. Data of Endovascularly Treated Patients

Endovascular treatment in 17 legs of 17 patients comprised eight recanalizations of complete occlusions of the superficial femoral artery and nine revascularizations of highly stenosed superficial femoral arteries.

Compared to day 0, the median ABI was found significantly increased on day one after endovascular treatment from 0.64 ± 0.25 to 0.91 ± 0.25 (*p* < 0.05).

Hyperspectral assessment revealed a significant increase of the NIR perfusion index in the angiosome of the dorsal pedal artery and peroneal artery. Although not statistically significant, the NIR perfusion index markedly increased in both the medial and lateral plantar artery. Compared to day 0, StO_2_ was also found increased in the angiosomes of the foot and the peroneal artery but only reached statistical significance in the medial and lateral plantar artery angiosomes (Table 3).

Although the ABI and both the NIR perfusion index and StO_2_ increased after the intervention, a significant correlation was only found for ABI and StO_2_ in the angiosome of the lateral plantar artery (R = 0.52, *p* = 0.032) and the peroneal artery (R = 0.54, *p* = 0.026), as well as for the NIR perfusion index in the angiosome of medial plantar artery (R = 0.54, *p* = 0.025) and the sural artery (R = 0.54, *p* = 0.042).

In comparison to surgically treated patients, the TWI also increased after endovascular treatment, but this effect was less significant (Table 3).

## 4. Discussion

This study demonstrates that HSI is feasible for detecting changes in skin oxygen saturation in the lower limb after surgical and endovascular treatment in patients with PAD (i). In addition, we show that the assessment of skin oxygen saturation is investigator-independent (ii) and not significantly affected by temperature (iii) or physical activity (iv).

HSI is a non-invasive, non-contact and non-ionizing technique that has gained more and more impact in clinical medicine in recent years. In visceral surgery, it is used to assess anastomosis in the upper [25] and lower gastrointestinal tract [26,27,28]. In neurosurgery, HSI can be used for intraoperative brain cancer targeting [29,30,31]. In reconstructive surgery, HSI is feasible for identifying cutaneous perforators for microvascular surgery [32] and for perioperative perfusion monitoring of free and pedicled flaps [33,34]. It was further established for assessment of wounds [35]. Here, it was shown, that HSI can be used to evaluate microcirculatory changes in diabetic foot ulcers and to predict clinical outcomes of wound healing [36,37]. The feasibility of assessing the presence and severity of PAD by means of HSI was initially shown in 2011 [16]. Our group recently proved that this technique can be used to improve the diagnosis of PAD [17]. Based on this, we now studied whether HSI is feasible for detecting changes in tissue oxygenation saturation after surgical or endovascular therapy in patients with PAD.

HSI allows the study of oxygenation saturation in superficial parts of the respective tissue at a depth of up to 3 mm based on penetration of near infrared (NIR) or visible light (StO_2_). In turn, HSI gives real-time, visually presented information on tissue oxygenation in the microcirculatory system. This is different to established techniques in vascular surgery, including the ABI, duplex ultrasound or angiographic methods, which only provide information on blood flow dynamics in perfused vessels of the macrocirculation, i.e., arteries and veins. With these techniques, no information can be obtained about the actual tissue perfusion or the local oxygen saturation and, thus, about the function of the microcirculation [38]. The contact-free analysis is another advantage compared to transcutaneous oximetry (tcpO_2_), ABI assessment or duplex ultrasound, which need a direct contact to the skin and are not suitable for perfusion assessment in painful wounds. Moreover, early-stage PAD especially might be missed by these methods since they only assess blood flow and not the effects on tissue oxygenation, which could be impaired first, as a sign of impaired microcirculation [1]. In addition, tissue perfusion is frequently impaired in patients with diabetes, who have a 10 to 20% higher prevalence of PAD, associated with both macro- and microcirculatory disorders, consecutive foot ulcers and the need for respective minor amputations [1]. In patients with diabetic foot syndrome, HSI could be used for annual follow-up, which is recommended by international and national guidelines [39,40]. The provided information on microcirculation could be added to the information on macrocirculation gained by established techniques. In this context, Horstick et al. studied tissue optical perfusion pressure in combination with ABI analysis in patients with PAD and showed that a combination of both techniques provides information on both macro- and microcirculation [1].

Routinely used as a non-invasive technique to locate vascular pathologies in macrocirculation, duplex ultrasound gives information on arterial blood flow dynamics, wall characteristics and location of stenosis or occlusion. However, its results depend on the experience of the user in conduction and interpretation of examination. In contrast, HSI does not require long-term training and experience for conduction and interpretation. This is likely due to the standardized parameter calculation with the integrated software. Like Chin et al., we found a non-significant inter-operator variability as a sign of objective, user-independent data acquisition with the TIVITA^®^ Tissue [16]. This is an important advantage for daily use and accounts for an objective, observer-independent analysis when using HSI in diagnosis of PAD. In this context, another advantage is the short time needed for data acquisition and quantification (approximately 16–20 s to take the picture and quantify the respective parameters). This is fast compared to the assessment of tcpO_2_ (more than 8 min) or the ABI (more than 3 min).

Tissue oxygenation saturation was studied by quantification of the NIR perfusion index and StO_2_ in seven angiosomes of the lower limbs. Angiosomes were first described in 1987 by Taylor and Palmer [18]. This concept is based on the finding that the perfusion of a part of the skin can be attributed to specific terminal arteries that supply the respective angiosome [18]. In the lower legs, four angiosomes (anterior tibial artery, sural artery, peroneal artery and posterior tibial artery) were described and three in the foot (dorsal pedal artery, the lateral plantar artery and the medial plantar artery). In vascular surgery, the angiosome concept was applied for direct revascularization of angiosomes with diabetic ulcers and non-diabetic wounds in patients with critical limb ischemia [3]. Especially for endovascular procedures, it was shown that direct revascularization of an artery referring to an angiosome with a non-healing wound can improve wound healing [41].

In this study, the angiosomes served for standardized execution of HSI analysis. We could show that surgical therapy significantly increased the NIR perfusion index on day three in two out of four angiosomes in the calf and in all three angiosomes of the foot. StO_2_ values increased as well in the most studied angiosomes but were not statistically significant. In contrast, in endovascularly treated patients, the NIR perfusion index increased as well in six out of seven angiosomes but not as significantly as after surgical treatment. In parallel, StO_2_ increased after endovascular therapy only in the angiosomes of the foot. One possible explanation for the less significant increase in both parameters could be the single measurement on day one after the intervention. This was due to the scheduled discharges of the patients on day one after the intervention. However, a longer follow-up after respective therapies should be considered for upcoming studies. Another explanation for the non-significant increase in some of the angiosomes might be the initially arteriosclerotic occlusion of the respective terminal arteries. Although these terminal arteries could be visualized in the preoperative/preinterventional imaging, a subgroup analysis on perfused and non-perfusion terminal arteries in correlation with the postoperative/postinterventional oxygenation values was not performed due to the small number of patients. The non-significant change in oxygenation saturation in some of the studied angiosomes might also be explained by the short time between revascularization and HSI, especially after endovascular therapy. As described in several studies, changes in perfusion may take weeks after respective therapy to become apparent [42]. This aspect further underlines the need for a longer follow-up in coming studies.

Despite tissue oxygenation saturation analysis, HSI also allows quantification of tissue water by means of the TWI. The TWI significantly increased in all studied angiosomes after surgical intervention. This reflects the increased tissue edema following ischemia reperfusion injury that is frequently seen in different manifestations after revascularization [43]. From the 16 studied legs, bypass implantation was performed in 12 cases of completely occluded iliac (three out of twelve) and superficial femoral arteries (nine out of twelve). This led to an immediate increase in blood flow into the chronic, inferiorly perfused limb and, therefore, induced an ischemia reperfusion injury with increased capillary leakage, formation of reactive oxygen species and subsequent tissue edema. Tissue edema detected by the TWI was less significant after endovascular procedures, which might be explained by the fact that, of 17 studied patients, only six had a recanalization of an occluded superficial femoral artery, but five patients got a stent-PTA due to high grade stenosis. Although leg perfusion was increased by this therapy, the gained additional blood flow was minor when compared to a bypass implantation. Consequently, the induced reperfusion injury can be assumed to be less significant, leading, in turn, to less tissue edema. Another reason for the postoperative increase in the TWI might be impaired lymph drainage due to surgical tissue trauma at the site of vascular access. In summary, the TWI could enable the physician to quantify edema formation before and after revascularization and might allow an indirect conclusion on ischemia-reperfusion injury.

Referring to the small number of patients in this study, it was not possible to perform subgroup analysis of all the studied angiosomes with respect to initial vascular status and the location of arterial occlusion or stenosis. This will be an important issue for coming studies because vascular anatomy varies widely in PAD, from short stenosis that limits blood flow to complete arterial occlusion. Furthermore, collateralization, which varies as well with respect to duration of PAD, physical activity of the patient and other factors also affect tissue oxygenation saturation. Formation of the collateral arteries due to ischemia-induced neoangiogenesis does not respect the borders of defined angiosomes and affects local tissue perfusion as well. In turn, microcirculation assessed by means of HSI in an angiosome can be normal although the corresponding artery is stenosed or even occluded.

Although HSI is actually used in different medical conditions, the limitations and potential effectors on this technique have to be considered as well. One variable affecting HSI is the skin’s melanin concentration [44,45]. However, all participants of this study were North European with comparable skin color. Due to the small number of studied patients, a separate analysis of sex, comorbidities or performed revascularization technique was not conducted.

The effect of temperature on HSI analysis was evaluated after a hot or cold foot bath. Neither heat or cold significantly affected the NIR perfusion index or StO_2_. However, both parameters slightly increased compared to baseline condition. For the warm footbath this effect can be explained by heat-induced vasodilatation. Compared to this, a cold foot bath induces vasoconstriction and, therefore, a decline in tissue oxygenation saturation. However, cold also leads to reduced metabolic activity of tissues and, therefore, reduced oxygen uptake. This might explain the slightly increase in the NIR perfusion index and StO_2_. In parallel, Goetze et al. found an improved detection rate of perforator vessels by cooling-induced skin reperfusion after three minutes of cooling [32].

## 5. Conclusions

In summary, HSI-based analysis of the NIR perfusion index and StO_2_ can be a useful, additional tool for both detection and monitoring of leg perfusion in patients with PAD in an examiner-independent, fast, non-contact way. As a consequence, a reduction of tissue oxygenation saturation parameters might lead to further vascular imaging to localize vascular pathologies that could be treated before significant tissue damage occurs. Although HSI is not intended to replace established techniques, it overcomes several disadvantages of the methods currently used to evaluate PAD. Extended studies with a larger cohort and a longer follow-up are needed to evaluate sensitivity and specificity. In this context, future studies should also address neuropathy as a potential effect on tissue oxygenation saturation changes after revascularization. All in all, this would also allow the study of the effects of the performed revascularization (local: angioplasty, thrombendarteriectomy; long-distance: bypass implantation, recanalization) on the tissue oxygenation saturation in respective angiosomes.

## Figures and Tables

**Table 1:** Characteristics of healthy volunteers (control group) and patients with peripheral artery disease.

	Control Group (*n* = 25)	PAD Patients (*n* = 37)
Sex (male/female)	21/4	30/7
Mean age (years)	26.4	64.9
Active smoking (%)	5 (20%)	23 (62%)
Arterial hypertension (%)	0%	27 (73%)
Diabetes mellitus (%)	0%	14 (38%)
Fontaine stadium		
IIa	/	5 (14%)
IIb	/	19 (51%)
III	/	6 (16%)
IV	/	7 (19%)

PAD: peripheral artery disease.

**Table 2:** Near infrared (NIR) perfusion index, StO_2_ and tissue water index (TWI) in the seven studied angiosomes before (day 0) the surgical revascularization and on the first and third postoperative day.

Angiosome	NIR Perfusion Index			StO_2_			TWI		
	Day 0	Day 1	Day 3	Day 0	Day 1	Day 3	Day 0	Day 1	Day 3
ATA	41 ± 7	42 ± 7	49 ± 9 *	44 ± 9	39 ± 7	42 ± 9	47 ± 5	48 ± 6	55 ± 9 *
PTA	42 ± 5	40 ± 10	47 ± 10	44 ± 7	38 ± 7 *	41 ± 8	46 ± 6	51 ± 10	56 ± 10 *
SA	41 ± 6	41 ± 7	44 ± 8	46 ± 7	39 ± 7 *	40 ± 8	46 ± 4	49 ± 8	53 ± 8
PA	41 ± 6	43 ± 8	47 ± 8 *	44 ± 7	40 ± 8	39 ± 8	50 ± 7	50 ± 8	56 ± 7 *
DPA	37 ± 7	43 ± 8 *	44 ± 9 *	36 ± 7	45 ± 11	40 ± 12	48 ± 9	49 ± 8	54 ± 8 *
MPA	51 ± 11	52 ± 9	57 ± 8 *	50 ± 14	57 ± 10	56 ± 12	51 ± 7	66 ± 10 *	61 ± 9 *
LPA	49 ± 11	53 ± 10	57 ± 9 *	52 ± 12	59 ± 11	59 ± 12	55 ± 7	66 ± 7 *	62 ± 7 *

Anterior tibial artery (ATA), posterior tibial artery (PTA), sural artery (SA), peroneal artery (PA), dorsal pedal artery (DPA), medial plantar artery (MPA) and lateral plantar artery (LPA). Data are given as mean ± SD. Skillings–Mack test. * *p* < 0.05 vs. day 0. *n* = 10–16.

**Table 3:** NIR perfusion index, StO_2_ and tissue water index (TWI) in the seven studied angiosomes before (day 0) and on the first day after endovascular treatment.

Angiosome	NIR Perfusion Index		StO_2_		TWI	
	Day 0	Day 1	Day 0	Day 1	Day 0	Day 1
ATA	38 ± 10	37 ± 11	40 ± 5	40 ± 8	48 ± 7	45 ± 5 *
PTA	39 ± 6	42 ± 8	40 ± 6	39 ± 7	46 ± 9	44 ± 6
SA	39 ± 7	40 ± 8	42 ± 7	42 ± 6	47 ± 9	44 ± 6
PA	41 ± 7	43 ± 8 *	41 ± 6	43 ± 9	50 ± 1	47 ± 8
DPA	36 ± 9	41 ± 11 *	36 ± 7	43 ± 12	47 ± 6	45 ± 5
MPA	50 ± 10	54 ± 8	46 ± 11	54 ± 1 *	52 ± 6	53 ± 7
LPA	50 ± 11	54 ± 7	49 ± 1	56 ± 1 *	55 ± 5	56 ± 6

Anterior tibial artery (ATA), posterior tibial artery (PTA), sural artery (SA), peroneal artery (PA), dorsal pedal artery (DPA), medial plantar artery (MPA) and lateral plantar artery (LPA). Data are given as mean ± SD. Wilcoxon signed-rank test. * *p* < 0.05 vs. day 0. *n* = 17.

## Data Availability

The data presented in this study are available on request from the corresponding author. The data are not publicly available.
